# When psychosis follows obsession – a schizo-obsessive disorder case report

**DOI:** 10.1192/j.eurpsy.2021.2106

**Published:** 2021-08-13

**Authors:** F. Pereira, P. Martins, J. Barros

**Affiliations:** Departamento De Psiquiatria E Saúde Mental, Unidade Local de Saúde do Nordeste, Porto, Portugal

**Keywords:** Schizo-Obsessive Disorder

## Abstract

**Introduction:**

The relation between obsessive-compulsive symptoms and psychosis presents in different ways. While obsessive-compulsive symptoms can present as prodromes of schizophrenia, or in overlap with psychotic phenomenology, a new clinical entity as been proposed as a subgroup of schizophrenia: the schizo-obsessive disorder.

**Objectives:**

The present review aims to emphasize the comorbility between schizophrenia and obsessive-compulsive disorder, bringing to light the importance of early detection and adequate treatment approaches.

**Methods:**

The authors describe the patient’s disease progression and discuss the longitudinal dynamics between obsessive-compulsive disorder and schizophrenia, providing a brief and updated literature overview.

**Results:**

The illustrated case addresses a 31-year-old male patient diagnosed with obsessive-compulsive disorder at the age of 16, who later developed delusional ideation compatible with a schizophrenia diagnosis. At the onset of disease, the patient developed obsessive-compulsive symptoms such counting and repetitive hand-washing rituals that later turned into sexual obsessions concerning homossexuality. Following his 25th birthday, the patient became increasingly disorganized with frequent agressive outbursts toward his family and the obsessive egodystonic ideation turned into delusional egosyntonic ideation. Over the years, the patient shows intermittent obsessive-compulsive behavior while sustaining schizophrenia symptoms, particularly the negative symptoms.

**Conclusions:**

Despite the controversy associated with the recently proposed new subgroup of schizophrenia, the schizo-obsessive disorder, we believe the patient described fits the diagnosis. Clinicians managing patients of schizophrenia should evaluate the patients thoroughly for presence of comorbid obsessive-compulsive symptoms/disorder and must take the same into account while managing the patients.

**Disclosure:**

No significant relationships.

